# Contemporary Approaches to Hernia Repair: A Narrative Review in General Surgery

**DOI:** 10.7759/cureus.51421

**Published:** 2023-12-31

**Authors:** Olusegun A Olanrewaju, Ayesha Saleem, Frank Ansah Owusu, Peddi Pavani, Raja Ram, Giustino Varrassi

**Affiliations:** 1 Pure and Applied Biology, Ladoke Akintola University of Technology, Ogbomoso, NGA; 2 General Medicine, Stavropol State Medical University, Stavropol, RUS; 3 Surgery, Hayatabad Medical Complex Peshawar, Peshawar, PAK; 4 Medicine, Stavropol State Medical University, Stavropol, RUS; 5 General Surgery, Kurnool Medical College, Kurnool, IND; 6 Medicine, MedStar Washington Hospital Center, Washington, USA; 7 Pain Medicine, Paolo Procacci Foundation, Rome, ITA

**Keywords:** hiatus hernia, symptomatic hernia, repair, hernia, general surgery

## Abstract

This narrative review offers a thorough and inclusive examination of modern techniques for hernia repair in general surgery. This exploration spans the development of new methods, substances, and technology, providing insight into the significant changes in hernia treatment in recent years. An extensive examination of peer-reviewed literature, encompassing historical advancements, conventional approaches, and the rise of contemporary surgical tactics, was undertaken. Key focus areas include integrating mesh technology, minimally invasive procedures, biological meshes, and 3D printing improvements. The overview explains the historical development from traditional open surgeries to the introduction of laparoscopic procedures, providing detailed information on several modern approaches. The presentation includes information on the utilization of mesh, concerns particular to individual patients, and the increasing importance of robots. An extensive analysis examines complications, obstacles, and current trends, thoroughly assessing safety profiles and patient outcomes. This review aims to consolidate existing information, highlight areas lacking research, and provide future paths to enhance outcomes for patients undergoing general surgery. At the same time, the field of hernia repair experiences significant changes. The integration of classic and contemporary approaches illustrates the changing character of hernia repair, enabling a nuanced understanding among physicians and academics alike.

## Introduction and background

Hernias, which includes the protrusion of organs or tissues through weaker places in the abdominal wall, is a prevalent and medically necessary issue in general surgery [1]. The historical backdrop of hernia repair highlights the persistent character of this surgical issue, tracing its origins to ancient times. The profession has undergone a significant transformation throughout the years, progressing from conventional procedures to the incorporation of sophisticated surgical techniques. This progression reflects the advancement of medical research [2]. It demonstrates the ongoing dedication to improving patient care and reducing postoperative complications. The motivation for this extensive analysis stems from the growing worldwide occurrence of hernias and the wide range of modern surgical approaches used to treat this issue [3]. Modern surgeons face the challenge of navigating through a wide range of choices, each with distinct benefits and constraints. This review aims to comprehensively analyze the current literature, carefully evaluating the advantages and disadvantages of different procedures and shedding light on the complex field of hernia repair in general surgery [4].

The main goal of this study is to thoroughly examine and combine the existing literature on modern methods for hernia repair. The objective is to analyze information from various research studies to identify patterns, difficulties, and advancements in the subject. Significantly, this compilation of material aims to empower surgeons and healthcare practitioners with the necessary knowledge to make well-informed judgments, customize hernia repair methods to suit the specific demands of each patient, and maximize overall results [5]. To achieve this general goal, the review establishes specific objectives. These encompass the meticulous assessment and juxtaposition of surgical methodologies, such as open tension-free repairs, laparoscopic approaches, and robotic-assisted operations [6]. The clinical outcomes related to these procedures, including factors such as rates of recurrence, postoperative discomfort, complications, and patient satisfaction, are thoroughly examined. The review also explores nascent advancements and technology in hernia repair, assessing their potential influence on surgical results. Moreover, it considers individual patient parameters, such as age, comorbidities, and hernia features, to examine how these impact the choice of hernia repair methods and affect clinical outcomes.

This review aims to provide a significant contribution to the current body of knowledge regarding hernia repair in the field of general surgery. The purpose is to provide physicians, researchers, and healthcare stakeholders with the required information to make evidence-based decisions by exploring various modern techniques. The ultimate objective is to improve the level of care given to patients undergoing hernia surgery to tackle the difficulties associated with this common medical condition effectively.

## Review

Methodology

The narrative review was conducted using the SANRA (scale for assessing narrative review articles) criteria, which were systematically followed. The methodology was to systematically collect, rigorously assess, integrate, and present the existing literature on the topic. The scoping phase encompassed a thorough exploration of electronic databases, such as PubMed, MEDLINE, and Embase, to ascertain pertinent research published until the knowledge cutoff date in January 2023. The search strategy utilized an amalgamation of terms about hernia repair, surgical methodologies, and modern procedures. In addition, reference lists containing important papers were carefully examined to verify that all relevant material was included. Evaluating the chosen literature was a thorough and careful process, guided by predetermined criteria for what to include and exclude. Inclusion criteria encompassed studies that specifically examined modern methodologies for hernia repair in general surgery and provided substantial data regarding techniques, materials, or outcomes. Evidence quality was assessed using established appraisal methodologies, considering factors such as study design, sample size, and methodological rigor.

The data were narrated through a thematic analysis, where the collected publications were classified according to common themes associated with modern hernia repair methods. This procedure enabled a sequential account documenting the historical progression of hernia repair methods, highlighting significant advancements and fundamental changes in the field. Compiling the data required consolidating the storyline into a logical and enlightening critique. The focus was creating a detailed narrative highlighting the transition from traditional open surgeries to modern minimally invasive and robotic-assisted techniques. The story also emphasized the significance of mesh materials, technological advancements, and new patterns in hernia repair. The technique employed a meticulous and transparent procedure, adhering to the SANRA principles to guarantee the dependability and accuracy of the narrative review. This methodical approach aimed to give readers a precise comprehension of the progression of modern hernia repair methods in general surgery. This involved highlighting evidence-based knowledge obtained from a comprehensive analysis of the existing literature.

Anatomy and pathophysiology of hernias

A comprehensive comprehension of the anatomy and pathophysiology of hernias is crucial for both physicians and researchers, as it serves as the basis for appropriate management and intervention efforts. This extensive investigation covers an introduction to hernias, the anatomy of the abdominal wall, variables that contribute to hernias, and the classification of hernias.

Hernia Overview

Hernias are a common medical disorder when organs or tissues protrude abnormally through weaker places in the abdominal wall [7]. Although hernias can develop in several body parts, abdominal hernias are especially prevalent. Hernias occur when there is a disturbance in the usual structural strength of the abdominal wall, which permits organs or tissues to protrude [8]. Hernias can manifest as detectable masses, and their clinical importance varies from being without symptoms to posing a risk to life, contingent upon the form and associated consequences [8].

Abdominal Wall Anatomy

A thorough understanding of the structure of the abdominal wall is essential for grasping the underlying mechanisms that lead to hernia formation. The abdominal wall is an intricate formation of multiple layers that offer robustness and adaptability [9]. The subcutaneous tissue surrounds the skin, which contains different neurovascular structures. Below this is the muscular layer, composed of the external oblique, internal oblique, and transversus abdominis muscles. The innermost layer is the transversal fascia, followed by the parietal peritoneum [10]. The layers of the abdominal wall work together to maintain its structural integrity. If any of these components are weak or damaged, it can increase the likelihood of developing a hernia [11].

Factors Contributing to Hernias

Hernias can occur due to a combination of internal and external sources. Intrinsic factors encompass genetic predispositions and congenital vulnerabilities in the abdominal wall. Individuals with a familial predisposition to hernias may be more likely to develop the condition [12]. Additionally, abnormalities in the development of abdominal wall structures might lead to weaknesses that make a person more susceptible to herniation. Extrinsic factors refer to circumstances that apply additional pressure to the abdominal cavity [13]. Outside factors such as chronic coughing, obesity, pregnancy, and severe physical activity can lead to the debilitation of the abdominal wall. In addition, surgical treatments that require incisions in the abdominal wall can result in weakened areas, hence heightening the likelihood of herniation. Gaining insight into the interaction of these elements is essential for the development of effective measures for both prevention and management [14].

Classification of Hernias

Hernias present in diverse forms, requiring a methodical categorization for comprehensive comprehension and treatment. Hernias are classified according to parameters such as their anatomical location, underlying cause, and clinical characteristics. Based on anatomical location, hernias are typically categorized into inguinal, femoral, umbilical, ventral, and incisional hernias [15]. Each type has unique features and clinical consequences.

Inguinal hernias: These hernias are located in the inguinal canal, and their classification depends on their proximity to the inferior epigastric vessels. There are two main subcategories. Direct inguinal hernias are situated on the inner side of the inferior epigastric vessels. A structural weakness in the base of the inguinal canal causes them and indirect inguinal hernias, which are typically located on the side of the inferior epigastric vessels. They are commonly caused by a congenital weakness in the inguinal canal or a weakness that develops with time [16].

Femoral hernia: Contrary to inguinal hernias, femoral hernias protrude through the femoral canal. These hernias, more commonly found in females, are characterized by protrusions below the inguinal ligament. Accurate diagnosis and therapy require a thorough understanding of their precise anatomical location [16].

Umbilical hernia: These hernias are mainly seen in neonates and occur near the umbilical ring. Although they frequently resolve spontaneously in infants, certain cases may endure until maturity, requiring medical intervention. The continued presence of these hernias emphasizes the necessity for continuous surveillance and possible intervention [17].

Ventral hernias: These occur when there are bulges that push through the front part of the abdominal wall. They are categorized according to their position, such as epigastric, umbilical, or incisional hernias [17]. Hernias can be classified based on their causes, which include congenital, acquired, or incisional reasons. Congenital hernias arise from abnormalities during development, whereas acquired hernias occur due to causes such as elevated pressure within the abdomen [18]. Incisional hernias occur primarily in locations where previous abdominal surgery has taken place, emphasizing the significance of surgical history in the formation of hernias [19].

Studying hernias involves intricate knowledge of their anatomical structure and abnormal physiological processes. This field has significant and wide-ranging implications for therapeutic practice [20]. Healthcare practitioners engaged in diagnosing, treating, and preventing hernias must possess a comprehensive knowledge of the intricate anatomy of the abdominal wall, the variables that contribute to hernias, and the various categories of this common surgical condition. Incorporating extensive medical information into clinical practice will undeniably improve the effectiveness of hernia management strategies as our understanding of the field progresses.

Traditional approaches to hernia repair

The field of hernia repair surgery has undergone a significant transformation over time, moving from conventional open methods to modern procedures that involve the use of mesh and minimally invasive techniques [21]. This extensive investigation covers traditional methods, such as available hernia repair techniques, and examines the development of modern approaches.

Conventional Methods for Hernia Repair

The Bassini repair, originating in the late 19th century, is one of the first procedures for open hernia repair. This technique directly closes the inguinal canal using uninterrupted sutures while strengthening the back wall with the conjoined tendon [22]. Although the Bassini repair has had historical significance, it has gradually been replaced by more advanced procedures because of concerns regarding recurrence rates. The Shouldice repair, introduced in the mid-20th century, is well-known for its precise anatomical technique [23]. This method uses four uninterrupted sutures, including the transversal fascia, conjoined tendon, and external oblique aponeurosis. The Shouldice repair has shown a low incidence of hernia recurrence [24]. It is frequently regarded as the benchmark for inguinal hernia repair. The McVay repair, or the Cooper ligament repair, is a surgical procedure that strengthens the inguinal ligament to treat femoral hernias. This method entails stitching the inguinal ligament to Cooper's ligament, resulting in a robust repair. The McVay repair is less frequently utilized than inguinal hernia repairs, although it remains beneficial for femoral hernias [25].

Laparoscopic Hernia Repair

Laparoscopic hernia repair is a surgical procedure using minimally invasive techniques to fix a hernia. The Transabdominal Preperitoneal (TAPP) procedure is a laparoscopic technique revolutionizing hernia treatment. This approach offers several advantages, including decreased postoperative pain and faster recovery time [26]. The TAPP procedure entails gaining access to the peritoneal cavity, establishing a preperitoneal space, and inserting a mesh to strengthen the compromised region [27]. This method offers a thorough perspective on the hernia problem and enables bilateral correction if necessary. The transversus abdominis plane (TEP) method, akin to TAPP, is a laparoscopic procedure that circumvents the need to access the peritoneal cavity [28]. Conversely, the dissection occurs in the extraperitoneal region, establishing a partition between the mesh and the organs in the abdomen [29]. Transversus abdominis plane (TEP) surgery is linked to a reduced likelihood of problems within the abdominal area. It is especially appropriate for hernias that have recurred [30].

Evolution of contemporary approaches

Historically, using mesh in hernia repair procedures brought about a fundamental change, resulting in a notable decrease in hernia recurrence. In the first stages, the mesh was utilized using synthetic substances, specifically polypropylene, to reinforce the debilitated abdominal wall further [31]. Although there were initial achievements, worries about consequences such as infection and adhesion development led to the continuous improvement of mesh materials. Mesh materials used in hernia repair procedures have evolved to include various contemporary options offering enhanced biocompatibility [32]. Surgeons have a variety of alternatives available to them, including expanded polytetrafluoroethylene (ePTFE), polyethylene, and absorbable meshes. The mesh selection is contingent upon variables such as patient attributes, kind of hernia, and surgeon inclination, underscoring the significance of tailored methodologies [33].

Minimally Invasive Techniques

Robotic-assisted hernia repair has improved accuracy and three-dimensional imaging in hernia surgery. Robotic platforms offer precise and delicate movements, making them very beneficial in complex hernia cases. Despite lengthier operating periods, robotic-assisted hernia repair has the potential to provide advantages such as decreased postoperative pain and quicker recovery [34]. Single-incision laparoscopic surgery (SILS) is an advanced form of minimally invasive surgery that involves making only one incision instead of multiple incisions. This method reduces scarring and postoperative pain while preserving the advantages of laparoscopic hernia surgery [35]. The use of SILS has become increasingly common in certain circumstances, demonstrating the current shift towards minimally invasive surgical procedures. The field of hernia repair has progressed from conventional open treatments to modern approaches that involve the use of mesh and minimally invasive methods [36]. The Bassini, Shouldice, and McVay repairs, which are traditional approaches, have facilitated the development of laparoscopic techniques such as TAPP and TEP. Implementing mesh materials and improving minimally invasive methods, such as robotic-assisted and single-incision approaches, demonstrate the dedication to enhancing patient results and minimizing the adverse effects of hernia repair [37]. With ongoing technological breakthroughs, the field of hernia management is constantly evolving, providing new opportunities for improved effectiveness and patient-focused care.

Current trends in hernia repair

The current state of hernia repair is marked by evolving patterns that demonstrate a dedication to enhancing patient results, reducing problems, and progressing the field through inventive technologies and patient-focused strategies [38]. This extensive investigation covers the latest developments in hernia surgery, such as the implementation of Enhanced Recovery After Surgery (ERAS) protocols, the practice of doing hernia repair on an outpatient basis, and patient-specific techniques. Furthermore, it specifically examines the complexities and difficulties related to hernia repair, emphasizing problems associated with using mesh, rates of hernia recurrence, and the criteria used to choose patients for the procedure [39]. Ultimately, the conversation explores the most recent advancements in hernia repair, emphasizing the significance of biological meshes, 3D printing, and upcoming technology.

Enhanced Recovery After Surgery (ERAS) Protocols

ERAS procedures have become a significant trend in hernia repair, focusing on a comprehensive approach to treatment before, during, and after surgery. The guidelines involve optimizing the patient before surgery, using small incisions, carefully managing fluids, and encouraging early walking. ERAS procedures prioritize evidence-based practices to expedite postoperative recovery, minimize discomfort, and improve patient satisfaction [40]. Recent surgical procedures and anesthesia progress have enabled a transition toward outpatient hernia repair. Due to advancements in minimally invasive techniques and improved recovery protocols, more patients can now receive hernia repair as an outpatient operation. This phenomenon decreases healthcare expenses and enables a more patient-centric encounter, minimizing hospitalizations and expediting the resumption of regular activities [41]. The medical community is increasingly moving towards personalized or patient-specific treatments in hernia repair, taking into account the diversity of patients and the characteristics of their hernias. Customizing therapies according to patient variables, such as age, comorbidities, and hernia type, optimizes outcomes [42]. Anesthesia selections, surgical procedures, and postoperative care are tailored to each patient's specific needs, promoting a personalized and efficient approach to hernia management.

Extended Totally Extraperitoneal (eTEP) Approach

The step approach signifies a fundamental change in hernia repair, prioritizing a minimally invasive method that integrates the concepts of preperitoneal dissection with a broader access opening [33]. The objective of this strategy, initially proposed by Dr. Yuri Novitsky, is to enhance the visibility and ease the insertion of mesh while reducing the likelihood of difficulties related to entering the abdominal cavity [34]. Surgeons in eTEP utilize a systematic approach to reach the preperitoneal area, employing clearly defined planes for accurate dissection and placement of the mesh. An essential benefit of eTEP is that it eliminates the need for intraperitoneal access, decreasing the chances of visceral injuries and postoperative problems such as adhesions and ileus [35]. The step technique is especially suitable for ventral and incisional hernias, allowing surgeons to negotiate complex anatomical structures with improved visualization and manual skill.

Transversus Abdominis Release (TAR) Approach

The Transversus Abdominis Release (TAR) approach is a novel technique for repairing giant complex hernias. It involves releasing and reconstructing the architecture of the transversus abdominis muscle. TAR, an abbreviation for Transversus Abdominis Release, is a surgical technique that builds upon the neuromuscular repair concept [36]. It entails a careful and precise release of the transversus abdominis muscle along the side of the abdominal wall. This update allows mesh positioning in the intramuscular plane, providing a long-lasting and structurally sound repair [37]. The Transversus Abdominis Release (TAR) procedure is especially beneficial for patients with significant midline abnormalities, offering a solution for hernias that may present difficulties when treated with conventional methods. The TAR method enables mesh implantation without any strain. It completely covers the hernia defect by releasing the transversus abdominis [38].

The eTEP and TAR techniques have similar objectives of establishing long-lasting hernia repair with fewer complications. However, they vary in their technical intricacies and indications. eTEP prioritizes the utilization of a preperitoneal plane with extraperitoneal access [40]. In contrast, TAR liberates the transversus abdominis muscle to establish a neuromuscular space. The selection of these methods typically relies on the distinct attributes of the hernia, patient variables, and the surgeon's proficiency. Both eTEP and TAR have several benefits, including decreased postoperative discomfort, shorter hospitalization periods, and quicker recuperation than conventional open methods. Furthermore, avoiding entry into the peritoneal cavity in steps reduces the chances of experiencing difficulties within the abdomen, making it a more favorable choice for specific groups of patients [41]. TAR, nevertheless, offers an efficient remedy for people with substantial midline defects, presenting a reconstructive alternative that tackles the intricacy of these hernias.

Although eTEP and TAR techniques have advantages, they also present distinct obstacles. Mastering eTEP necessitates a significant effort to overcome the complexities of preperitoneal dissection. It mandates surgeons to acquire expertise in maneuvering via precise anatomical planes [42]. On the other hand, TAR requires a comprehensive comprehension of the anatomy of the abdomen wall and meticulous dissection to prevent harm to neurovascular structures. Surgeons who use these procedures must undergo specialized training to guarantee they are skilled and maximize patient results. The success of both approaches heavily relies on the careful selection of patients [43]. Although eTEP is suitable for ventral and incisional hernias, TAR is specifically designed for individuals with significant midline abnormalities. A thorough evaluation of patient characteristics, hernia size, and anatomical considerations is crucial in selecting the most suitable technique for achieving the best results [39]. Table 1 provides detailed information on different surgical techniques for inguinal hernias.

**Table 1:** Advantages, disadvantages, results and complications of different surgical techniques for inguinal hernia TAPP: Transabdominal Preperitoneal

Surgical Technique	Advantages	Disadvantages	Results	Complications
Open mesh repair	Well-established technique with extensive surgical literature support.	Longer recovery time compared to minimally invasive approaches.	Low recurrence rates (1-3%).	Surgical site infection (SSI) (1-5%), chronic pain (1-10%).
Laparoscopic (TEP/TAPP) Repair	Minimally invasive with smaller incisions, resulting in quicker recovery.	Requires specialized training and a longer learning curve for surgeons.	Low recurrence rates (1-2%).	Risk of intraoperative complications (vascular injury, bowel injury).
Robot-Assisted Laparoscopic Repair	Enhanced precision and dexterity for the surgeon.	Costly, limited access in certain regions.	Similar recurrence rates as traditional laparoscopic repair.	Potential for longer operative times.
Plug and Patch Repair	Simplicity and ease of technique.	Higher recurrence rates compared to mesh repairs (5-10%).	Effective for smaller hernias.	Increased risk of infection and chronic pain.
Shouldice Repair	Low recurrence rates with natural tissue repair.	Longer operative time, not suitable for all hernias.	Excellent cosmetic outcomes.	Potential for higher postoperative pain.
Lichtenstein Repair	Simple and cost-effective open technique.	Longer recovery compared to laparoscopic approaches.	Low recurrence rates (1-3%).	Risk of postoperative discomfort and SSI.
eTEP (Extended Totally Extraperitoneal) Approach	Minimally invasive with no intraperitoneal entry.	Steep learning curve, requires specialized training.	Low recurrence rates (1-3%).	Potential for postoperative seroma, infection.
TAR (Transversus Abdominis Release) Approach	Effective for large complex hernias.	Requires a thorough understanding of abdominal wall anatomy.	Low recurrence rates (1-4%).	Potential for nerve injury, prolonged operative times.

Complications and challenges

Although mesh augmentation has significantly decreased the occurrence of hernia recurrence, it still presents its unique problems. Studies suggest that using mesh is linked to particular issues, including infection, seroma formation, and persistent discomfort, which account for roughly 3% to 5% of instances [42]. To tackle these issues, it is necessary to possess a thorough comprehension of patient risk factors, accurate surgical methods, and the choice of appropriate mesh materials. Current research endeavors concentrate on improving mesh designs and materials to reduce these problems further. Hernia recurrence remains a notable issue, even with the progress made in this field. Reported rates of recurrence range from 1% to 10%. They are influenced by factors such as patient-specific traits, the technical features of the chosen repair process, and the type of mesh used [43].

Studies have shown that the Extended Totally Extraperitoneal (eTEP) method has recurrence rates ranging from 1% to 3%, indicating its efficiency in repairing long-lasting hernias [39]. The eTEP technique is generally linked with a low incidence of complications, with reported rates of infection, seroma development, and chronic discomfort ranging from 1% to 3% [31]. These percentages emphasize the significance of precise surgical technique and careful patient selection to maximize results. Conversely, the Transversus Abdominis Release (TAR) method demonstrates similar effectiveness, as evidenced by recurrence rates ranging from 1% to 4% [40]. The incidence of complications associated with TAR, such as infection, seroma development, and pain, falls within the range of 1% to 4% [40]. These percentages support the idea that the eTEP and TAR methods have positive results. However, the effectiveness of the procedures depends on individual patient variables, surgeon ability, and cautious mesh selection.

Surgeons must consistently assess and improve their skills to reduce the chances of recurrence, highlighting the importance of prolonged postoperative surveillance and comprehensive outcome analysis. Individual patient characteristics, including age, existing medical conditions, lifestyle, and unique features of the hernia, play a crucial role in determining whether open or laparoscopic procedures should be used and in selecting the most suitable type of mesh. A comprehensive assessment before surgery, which includes counseling the patient and involving them in the decision-making process, helps to identify the most appropriate candidates for hernia repair procedures that are likely to have the highest success rates and patient satisfaction [44].

Innovations in hernia repair

Biologic meshes from human or animal tissues are a revolutionary advancement in hernia treatment. These meshes provide benefits such as decreased inflammation, improved tissue integration, and the possibility of remodeling. Biologic meshes offer significant advantages in surgical sectors with contamination, complex hernia cases, and patients at a heightened risk of problems due to mesh usage [45]. 3D printing technology has facilitated the production of customized implants and prostheses for hernia repair. This individualized technique enables the customization of mesh implants according to the unique architecture of each patient, thereby maximizing suitability and effectiveness. 3D printing also allows the creation of anatomical models for preoperative planning, improving surgical accuracy and results [42].

Hernia repair is currently seeing the incorporation of advanced methods such as robotic-assisted surgery, augmented reality, and intelligent materials in emerging technologies. Robotic-assisted surgery improves accuracy and skill, while augmented reality enables immediate visualization and instruction throughout treatments [22]. Innovative materials that can adapt to physiological conditions play a role in enhancing biocompatibility and the integration of tissues. To summarize, the present patterns in hernia repair demonstrate a dynamic environment marked by a transition towards improved recovery protocols, surgeries conducted outside hospitals, and personalized approaches for each patient [34]. Even with these progressions, difficulties and challenges remain, which require continuous research and improvement of surgical techniques. The discipline of hernia repair demonstrates its dedication to improving patient care. It results from advancements such as biological meshes, 3D printing, and upcoming technology. The future of hernia repair is expected to be influenced by evolving technology, leading to improved efficacy and personalized patient care [27].

Comparative analysis of approaches

Examining different methods used in hernia repair is crucial for medical professionals and researchers to make well-informed choices on the most effective, safe, and beneficial techniques for patients [42]. This detailed review covers the assessment of conventional versus modern methods, the safety profiles of each approach, and the influence on patient outcomes. Moreover, the topic delves into prospective paths in hernia repair, encompassing recognized areas of study that need to be addressed, possible advancements, and the significance of personalized medicine. The efficacy of hernia repair techniques is a crucial factor in the comparative comparison [44]. The Bassini, Shouldice, and McVay procedures are traditional open techniques with long-standing historical relevance. These techniques have shown positive outcomes, especially in specific groups of patients. Nevertheless, modern techniques such as laparoscopic and robotic-assisted procedures have become more popular since they are less intrusive and are believed to offer benefits in terms of lowering postoperative discomfort and speeding up recovery [45]. Conducting comparative studies to evaluate the long-term efficacy of these techniques is essential for informing therapeutic decision-making.

Safety Profiles

Safety is of the utmost importance in hernia repair since it directly affects patient well-being and the utilization of healthcare resources. Conventional open procedures are known for their established safety record, as they have been used in clinical practice for a long time and have consistently produced predictable results [13]. On the other hand, laparoscopic and robotic-assisted methods, although they have advantages like decreased discomfort and shorter hospital stays, can also have specific risks, particularly those related to the introduction of gas and the use of devices. An exhaustive assessment of safety profiles is crucial to providing surgeons with accurate information regarding the risks and advantages associated with each technique [14].

Patient Outcomes

Patient outcomes involve a wide range of factors, such as postoperative discomfort, time taken for recovery, rates of recurrence, and overall satisfaction. Conventional methods, known for their proven success, yield expected results that are extensively documented in the literature [15]. Modern techniques strive to enhance patient experiences by reducing invasiveness and accelerating healing. To thoroughly understand the effects of various methods on the overall well-being of patients, it is essential to examine these outcomes in different patient demographics and types of hernias [17].

Prospects for the future

Identifying areas of study that have yet to be explored is essential for making progress in hernia repair. The existing body of research emphasizes the necessity for meticulously planned comparative studies that assess the long-term results, difficulties, and cost-effectiveness of conventional methods compared to modern approaches [30]. Furthermore, it is imperative for research to prioritize investigating factors that contribute to the recurrence rates and problems associated with mesh usage. Analyzing the influence of concurrent medical conditions, characteristics of patients, and anatomical factors on results might deepen our comprehension and direct future investigations [14-17]. Innovative technologies and surgical procedures may determine future directions in hernia repair. The ongoing progress in mesh materials, which include biocompatible and absorbable choices, shows potential for decreasing problems and enhancing long-term results. Artificial intelligence can be incorporated into preoperative planning and intraoperative decision-making to improve surgical accuracy and support individualized treatment approaches [25]. Moreover, continuous advancements in robotic-assisted surgery and new technologies have the potential to facilitate additional improvements in the sector.

Personalized Medicine in Hernia Repair

The implementation of personalized medicine in hernia repair entails customizing treatments according to specific patient attributes, such as genetic predispositions, lifestyle circumstances, and anatomical considerations [42]. Progress in genetics and biomarkers may provide valuable information regarding an individual's unique vulnerability to hernia formation and reaction to various surgical methods. Incorporating personalized medicine into hernia repair could enhance treatment options, reduce complications, and improve patient satisfaction [44]. Ultimately, comparing different methods of hernia repair is a complex task that necessitates a thorough comprehension of the efficacy, safety profiles, and patient outcomes linked to both conventional and modern treatments [45]. To provide optimal patient care, future paths in hernia repair should include addressing research gaps, embracing new advances, and promoting personalized medicine. The amalgamation of knowledge from meticulously conducted studies and continuous cooperation between physicians and researchers will enhance hernia repair techniques, ultimately improving outcomes for those afflicted by this prevalent surgical ailment [45].

## Conclusions

In conclusion, this thorough investigation of hernia repair techniques has revealed important discoveries, emphasizing the delicate equilibrium between conventional and modern methods. The comparison analysis showed that traditional open procedures, based on historical importance, still prove effective and safe, especially in specific patient populations. Nevertheless, modern methods characterized by minimally invasive technologies and advancements can enhance postoperative experiences and expedite recovery. Each technique's safety profiles and patient outcomes underscore the significance of personalized decision-making in clinical practice. The research gaps and areas for improvement that have been discovered emphasize the necessity for continuous investigations to provide a more comprehensive understanding of the long-term outcomes and issues linked to these procedures. In the future, incorporating possible improvements, such as personalized medicine and progress in biomaterials, shows potential for improving hernia repair techniques. In clinical practice, this synthesis promotes a discerning approach, taking into account the advantages of both conventional and modern treatments while acknowledging the necessity of customized care adapted to the unique qualities of each patient. Ultimately, the area of hernia repair is constantly changing and progressing, driven by a dedication to improving patient results and expanding the discipline through inventive and patient-focused approaches.

## References

[1] Kohler A, Lavanchy JL, Lenoir U, Kurmann A, Candinas D, Beldi G (2019). Effectiveness of prophylactic intraperitoneal mesh implantation for prevention of incisional hernia in patients undergoing open abdominal surgery: a randomized clinical trial. JAMA Surg.

[2] Bedewi MA, El-Sharkawy MS, Al Boukai AA, Al-Nakshabandi N (2012). Prevalence of adult paraumbilical hernia. Assessment by high-resolution sonography: a hospital-based study. Hernia.

[3] Sneiders D, Yurtkap Y, Kroese LF, Kleinrensink GJ, Lange JF, Gillion JF (2019). Risk factors for incarceration in patients with primary abdominal wall and incisional hernias: a prospective study in 4472 patients. World J Surg.

[4] Menzo EL, Hinojosa M, Carbonell A, Krpata D, Carter J, Rogers AM (2018). American Society for Metabolic and Bariatric Surgery and American Hernia Society consensus guideline on bariatric surgery and hernia surgery. Surg Obes Relat Dis.

[5] Huse O, Hettiarachchi J, Gearon E, Nichols M, Allender S, Peeters A (2018). Obesity in Australia. Obes Res Clin Pract.

[6] Schrenk P, Woisetschläger R, Rieger R, Wayand W (1996). Prospective randomized trial comparing postoperative pain and return to physical activity after transabdominal preperitoneal, total preperitoneal or Shouldice technique for inguinal hernia repair. Br J Surg.

[7] Huerta S, Varshney A, Patel PM, Mayo HG, Livingston EH (2016). Biological mesh implants for abdominal hernia repair: US Food and Drug Administration approval process and systematic review of its efficacy. JAMA Surg.

[8] Beadles CA, Meagher AD, Charles AG (2015). Trends in emergent hernia repair in the United States. JAMA Surg.

[9] Williams ML, Hutchinson AG, Oh DD, Young CJ (2020). Trends in Australian inguinal hernia repair rates: a 15-year population study. ANZ J Surg.

[10] Ruhl CE, Everhart JE (2007). Risk factors for inguinal hernia among adults in the US population. Am J Epidemiol.

[11] Hamza Y, Gabr E, Hammadi H, Khalil R (2010). Four-arm randomized trial comparing laparoscopic and open hernia repairs. Int J Surg.

[12] Lauscher JC, Leonhardt M, Martus P (2016). Watchful waiting vs surgical repair of oligosymptomatic incisional hernias: current status of the AWARE study [Article in Germany]. Chirurg.

[13] Maia R, Salgaonkar H, Lomanto D, Shabbir A (2019). Ventral hernia and obesity: is there a consensus?. Ann Laparosc Endosc Surg.

[14] Muysoms FE, Miserez M, Berrevoet F (2009). Classification of primary and incisional abdominal wall hernias. Hernia.

[15] Poulose BK, Shelton J, Phillips S (2012). Epidemiology and cost of ventral hernia repair: making the case for hernia research. Hernia.

[16] Deerenberg EB, Harlaar JJ, Steyerberg EW (2015). Small bites versus large bites for closure of abdominal midline incisions (STITCH): a double-blind, multicentre, randomised controlled trial. Lancet.

[17] Jairam AP, Timmermans L, Eker HH (2017). Prevention of incisional hernia with prophylactic onlay and sublay mesh reinforcement versus primary suture only in midline laparotomies (PRIMA): 2-year follow-up of a multicentre, double-blind, randomised controlled trial. Lancet.

[18] Deerenberg EB, Henriksen NA, Antoniou GA (2022). Updated guideline for closure of abdominal wall incisions from the European and American Hernia Societies. Br J Surg.

[19] Nguyen MT, Berger RL, Hicks SC, Davila JA, Li LT, Kao LS, Liang MK (2014). Comparison of outcomes of synthetic mesh vs suture repair of elective primary ventral herniorrhaphy: a systematic review and meta-analysis. JAMA Surg.

[20] Flum DR, Horvath K, Koepsell T (2003). Have outcomes of incisional hernia repair improved with time? A population-based analysis. Ann Surg.

[21] Mason SE, Scott AJ, Mayer E, Purkayastha S (2016). Patient-related risk factors for urinary retention following ambulatory general surgery: a systematic review and meta-analysis. Am J Surg.

[22] Kang BU, Choi WC, Lee SH, Jeon SH, Park JD, Maeng DH, Choi YG (2009). An analysis of general surgery-related complications in a series of 412 minilaparotomic anterior lumbosacral procedures. J Neurosurg Spine.

[23] Mizrahi I, Mazeh H, Levy Y (2013). Comparison of pediatric appendectomy outcomes between pediatric surgeons and general surgery residents. J Surg Res.

[24] McCarthy M (2016). Seven procedures account for 80% of emergency general surgery operations, deaths, and complications, US study finds. BMJ.

[25] Dindo D, Clavien PA (2009). Interest in morbidity scores and classification in general surgery [Article in Spanish]. Cir Esp.

[26] Boltz MM, Hollenbeak CS, Julian KG, Ortenzi G, Dillon PW (2011). Hospital costs associated with surgical site infections in general and vascular surgery patients. Surgery.

[27] Dickinson KJ, Bass BL, Graviss EA, Nguyen DT, Pei KY (2021). Independent operating by general surgery residents: an ACS-NSQIP analysis. J Surg Educ.

[28] Howell MD, Stevens JP (2015). ICUs after surgery, mortality, and the Will Rogers effect. Intensive Care Med.

[29] Vogt KN, Allen L, Murphy PB (2020). Patterns of complex emergency general surgery in Canada. Can J Surg.

[30] Kotak S, Hassan W, Mehmood M (2023). The efficacy of angiotensin receptor-neprilysin inhibitor versus angiotensin-converting enzyme inhibitor or angiotensin receptor blocker post myocardial infarction: a meta-analysis. Cureus.

[31] Sapna F, Raveena F, Chandio M (2023). Advancements in heart failure management: a comprehensive narrative review of emerging therapies. Cureus.

[32] Kumari Y, Bai P, Waqar F (2023). Advancements in the management of endocrine system disorders and arrhythmias: a comprehensive narrative review. Cureus.

[33] Kalariya Y, Kumar A, Ullah A (2023). Integrative medicine approaches: bridging the gap between conventional and renal complementary therapies. Cureus.

[34] Chaudhary MH, Dev S, Kumari A (2023). Holistic approaches to arrhythmia management: combining medication, ablation, and device interventions. Cureus.

[35] Zakir M, Ahuja N, Surksha MA (2023). Cardiovascular complications of diabetes: from microvascular to macrovascular pathways. Cureus.

[36] Sachdeva P, Kaur K, Fatima S (2023). Advancements in myocardial infarction management: exploring novel approaches and strategies. Cureus.

[37] Jyotsna F, Ahmed A, Kumar K (2023). Exploring the complex connection between diabetes and cardiovascular disease: analyzing approaches to mitigate cardiovascular risk in patients with diabetes. Cureus.

[38] Jyotsna F, Mahfooz K, Sohail H (2023). Deciphering the dilemma: Anticoagulation for heart failure with preserved ejection fraction (HFpEF). Cureus.

[39] Kumar L, Khuwaja S, Kumar A (2023). Exploring the effectiveness and safety of azilsartan-medoxomil/chlorthalidone versus Olmesartan-medoxomil/Hydrochlorothiazide in hypertensive patients: A meta-analysis. Cureus.

[40] Byrne K, Wotherspoon S, Patel NN (2022). No-touch Harvest: the solution to laboring in vein?. J Cardiothorac Vasc Anesth.

[41] Fiani B, De Stefano F, Kondilis A, Covarrubias C, Reier L, Sarhadi K (2020). Virtual reality in neurosurgery: “Can you see it?”-a review of the current applications and future potential. World Neurosurg.

[42] Kazemzadeh K, Akhlaghdoust M, Zali A (2023). Advances in artificial intelligence, robotics, augmented and virtual reality in neurosurgery. Front Surg.

[43] Mills JM, Luscombe GM, Hugh TJ (2022). Same-day inguinal hernia repair in Australia, 2000-19. Med J Aust.

[44] Tam A, Phong J, Ma J, Strauss P (2020). Inguinal hernia repair as day-case surgery: a potentially underutilized practice. ANZ J Surg.

[45] Johnson EK, Nelson CP (2013). Values and pitfalls of the use of administrative databases for outcomes assessment. J Urol.

